# Supplementary material for: Comparative GWAS using global and Indian Reference Panels reveals non-coding drivers of COVID-19 severity and mortality
Source: PLoS Negl Trop Dis. 2026 Mar 3;20(3):e0014020. doi: 10.1371/journal.pntd.0014020 (PMC12956133; doi:10.1371/journal.pntd.0014020)

S1 Fig. Population Stratification visualized by 3D PCA: 3D PCA revealed that none of the COVID-19 samples exhibited a Z-score greater than 6, and the samples clustered with the South Asian population, indicating no major outliers.

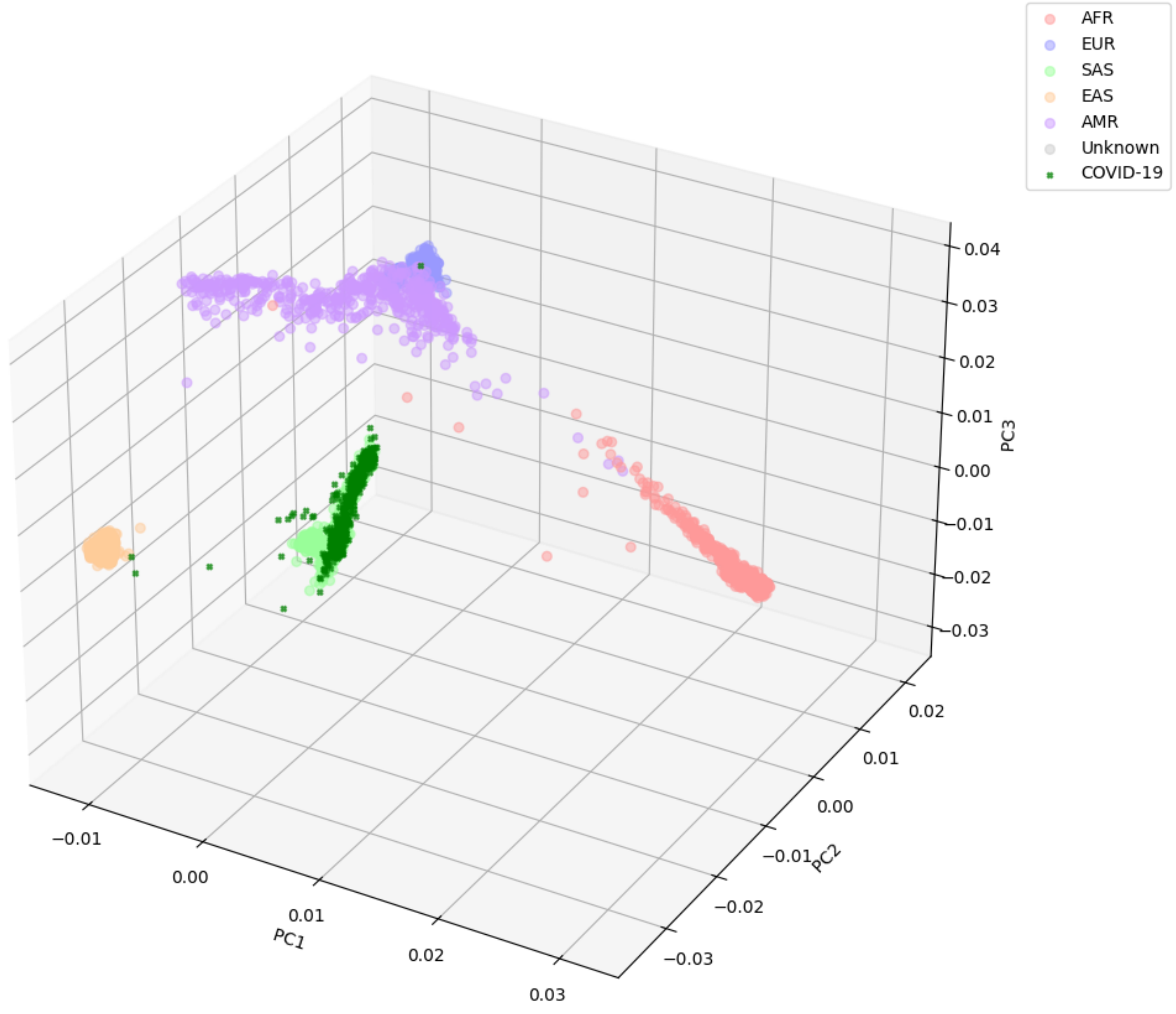

Supplement: S1 Fig — 3D PCA revealed that none of the COVID-19 samples exhibited a Z-score greater than 6, and the samples clustered with the South Asian population, indicating no major outliers. (PDF) [file pntd.0014020.s001.pdf]
