# Supplementary material for: Comparative GWAS using global and Indian Reference Panels reveals non-coding drivers of COVID-19 severity and mortality
Source: PLoS Negl Trop Dis. 2026 Mar 3;20(3):e0014020. doi: 10.1371/journal.pntd.0014020 (PMC12956133; doi:10.1371/journal.pntd.0014020)
Supplement: Attachment — Submitted filename: Reviwe comment.docx [file pntd.0014020.s008.docx]

The description of the post-QC dataset is generally clear and provides useful summary statistics regarding sample and variant retention. However, several clarifications and improvements would strengthen this section:

1. **Population Structure Analysis**
   The PCA results are described succinctly, but the text could clarify:
   - How population reference samples were incorporated (e.g., merged directly with study samples or projected).
   - The proportion of variance explained by PC1 and PC2.
   - Whether any population outliers identified in PCA were removed prior to analysis.
2. **Ancestry Consistency**
   The statement that the cohort was “ancestrally consistent” is reasonable based on clustering with the SAS super-population. However, since there is some overlap into EAS, please clarify whether this overlap is expected (e.g., due to regional admixture) and whether these individuals were retained or removed.

The section describing the chromosome 13 signal and the novel chromosome 2 region provides an informative overview of the mortality-specific association findings. The consistency of the Chr13 signal across two independent imputation panels is particularly notable. However, several aspects would benefit from further clarification or expansion:

1. **Statistical Strength of Associations**
   The text refers to these variants as “suggestive,” but does not state their p-values, confidence intervals, or effect sizes. Including these metrics is essential for assessing the robustness of the findings. Clarify whether any associations approach genome-wide significance or remain modest.
2. **Small Mortality Sample Size**
   The mortality group (N = 66) is relatively small compared to the recovered group. Discuss how this limited sample size may reduce power, increase false-positive risk, or inflate effect size estimates.
3. **Population Structure Considerations**
   Since ancestry differences between recovered and mortality groups could confound associations, it would be appropriate to mention whether covariates (e.g., PCs, age, comorbidities) were included in the regression model.

Interesting potential shown a regulatory locus on chromosome 2, and the integration of LD structure, genomic context, and functional plausibility is a strong aspect of the analysis. However, several points could be clarified or strengthened:

1. **Statistical Strength and Significance**
   While the LD block and suggestive SNPs are clearly described, the paragraph does not provide their p-values or effect sizes. Inclusion of these details is essential for assessing how robust and biologically meaningful these associations may be. It would also be helpful to clarify whether any SNPs approach genome-wide significance or whether the findings remain fully exploratory.
2. **LD Block Interpretation**
   The LD structure is well described, but it would be useful to mention:
   – whether the LD block is unique to this study cohort or also observed in external South Asian populations (e.g., 1000G SAS).
   – whether recombination hotspots or regulatory elements are known in this region.
   This contextualization would strengthen the argument for biological relevance.
3. **Non-coding RNA Focus**
   The paragraph correctly notes that no coding genes overlap with these SNPs, suggesting regulatory roles. However, it would be valuable to discuss whether AC007100.1 or MIR4432HG show differential expression in COVID-19 or in endothelial dysfunction contexts based on existing literature or transcriptomic datasets.
4. **Relevance to COVID-19 Pathophysiology**
   The linkage to endothelial dysfunction is biologically plausible given known mechanisms of severe COVID-19. Nonetheless, adding a brief explanation of how FGFBP1 dysregulation relates specifically to vascular pathology, hyperinflammation, or thrombosis would strengthen the interpretive flow.
