# Supplementary material for: Comparative GWAS using global and Indian Reference Panels reveals non-coding drivers of COVID-19 severity and mortality
Source: PLoS Negl Trop Dis. 2026 Mar 3;20(3):e0014020. doi: 10.1371/journal.pntd.0014020 (PMC12956133; doi:10.1371/journal.pntd.0014020)
Supplement: Attachment — Submitted filename: Response to Reviewers_PNTD-D-25-01448.docx [file pntd.0014020.s010.docx]

**Response to Reviewers**

Reviewer #1: This study investigates the genetic differences among people in India that may affect how severely COVID-19 impacts them and their chances of survival. The authors suggest that key indigenous genetic signals influencing disease severity and outcomes might be overlooked if imputation is based on a reference panel that underrepresents the studied population. The study found some loci were shared between the IndiGen and 1KGenomes panels, while others were unique to the Indian population. The manuscript is well written, with data analysed systematically and thoroughly.

We are thankful to the Reviewer for summarizing the findings and appreciating our efforts.

R1-Q1: Line 492: “…samples were collected from patients with confirmed SARS-CoV-2 infection...” Please specify how the infections were confirmed.

We thank the reviewer for this query. SARS-CoV-2 infection was confirmed by real-time RT-PCR at the hospital upon patient visiting the hospital for the first time. This information is now clarified in the Methods section of the revised manuscript.

R1-Q2: To ease readers' understanding, consider using “Mild vs Severe” instead of “Control vs Case”. Justification: “Control” generally means no disease or infection. By using “Mild vs Severe”, readers are reminded throughout the manuscript that the subjects were all confirmed infected with SARS-CoV-2 and exhibited clinical symptoms.

We thank the reviewer for this valuable insight. We understand the reviewer's concern that the term ‘*control*’ could be interpreted as referring to uninfected individuals. In this study, as mentioned by you, all patients were RT-PCR confirmed SARS-CoV-2 infected individuals, and the terms were used to denote COVID-19 severity categories, with ‘control’ referring to mild disease and ‘*case’* referring to moderate to severe disease, as clearly defined in the patients' stratification described in the **Methods** section. To address this concern and further improve clarity for readers, we have reiterated this severity definition at the first occurrence in the **Results** section, as well as in the **figure legends and panel labels** (e.g., *Control [Mild] vs Case [Moderate + Severe]*), while retaining the original terminology for consistency across the analyses. We reciprocate your support on this.

R1-Q3: Consider replacing “Recovered vs Mortality” with “Recovered vs Deceased” instead in the figures, tables, and text.

We thank the reviewer for this suggestion. We agree that the term deceased provides better clarity when referring to patients’ outcomes. Accordingly, we have now replaced “Mortality” with “Deceased” throughout the revised manuscript, including the main text, figures, and tables to improve readability and consistency.

R1-Q4: Were the key respiratory parameters listed in the supplementary table and used in determining the patient subgroups obtained at the point of admission?

Yes. All key respiratory parameters used for patient stratification and reported in the supplementary table were recorded at the time of hospital admission. This has now been clarified in the methods section of the revised manuscript.

R1-Q5: Different SARS-CoV-2 variants are also known to affect disease severity and clinical outcomes. It is suggested that a brief discussion of this is included in the manuscript.

We thank the reviewer for this valuable suggestion. We agree that SARS-CoV-2 variants of concern may influence disease severity and clinical outcomes. The present study is based on the samples collected between a single relatively narrow time window of May-September 2020, and it corresponds to the initial cases of COVID-19 in India, before the emergence of the delta variant. (https://doi.org/10.5005/jp-journals-10071-24042). As the cohort was not collected across multiple COVID-19 pandemic phases, viral lineage heterogeneity is expected to be limited during the time.

Accordingly, we have now added a brief discussion acknowledging the potential contribution of viral variants to clinical heterogeneity. We also emphasize the importance of future studies that integrate host and viral genomic data.

**The following statement has been added to the discussion section of the manuscript.**

“Alongside host genetic factors, it is also recognized that circulating SARS-CoV-2 variants of concern (VOC) have been reported to differ in their clinical severity and outcomes, highlighting the potential contribution of viral diversity to clinical heterogeneity. In the present study, viral lineage information was not available for stratified analysis as the study was specifically designed to investigate host genetic susceptibility using a uniform clinical classification criterion across patients recruited during a single time window corresponding to the first wave of COVID-19 in India. As the samples were not collected across multiple epidemic phases, variant-driven heterogeneity is expected to be limited. Future studies integrating host genotype data with viral genomic information will be important to further disentangle host and virus driving contributors to COVID-19 severity. (<https://doi.org/10.1016/s0140-6736(22)00462-7>, <https://doi.org/10.1016/j.ijregi.2024.03.003>, <https://doi.org/10.1371/journal.pcbi.1012469>)”

**Reviewer #2:**

R2-Q1: The description of the post-QC dataset is generally clear and provides useful summary statistics regarding sample and variant retention. However, several clarifications and improvements would strengthen this section:

1. **Population Structure Analysis**
 The PCA results are described succinctly, but the text could clarify:

o	How population reference samples were incorporated (e.g., merged directly with study samples or projected).

o	The proportion of variance explained by PC1 and PC2.

o	Whether any population outliers identified in PCA were removed prior to analysis.

We thank the reviewer for these detailed and constructive suggestions. We have revised the manuscript to address each concern sequentially. The suggested changes are as follows:

**Materials and Methods: Population Structure and Outlier Detection**

“Population structure was assessed using Principal Component Analysis (PCA) after genotype-level quality control. To assess global ancestry composition, we performed a two-dimensional (2D) PCA by projecting study samples directly onto reference populations from the 1000 Genomes Project, enabling direct comparison with major continental populations **(Fig.1B-i)**. This analysis demonstrated predominant clustering of the cohort with the South Asian (SAS) super-population. To enhance resolution and identify potential extreme ancestry deviations, a three-dimensional PCA (3D) was also examined using a Z-score threshold ≥ 6, which did not reveal any additional population outliers **(Supplementary Fig. S1)**. In addition, a within-cohort PCA restricted to the GSA dataset was performed to identify potential technical or extreme population outliers independent of the global reference structure. This analysis identified two clear outliers, which were removed before imputation and downstream association analyses **(Fig. 1B-ii and 1B-iii).** The top 10 principal components (PC1-PC10) derived from the PCA were included as covariates in all the association analyses.”

**Results: Quality review and analysis**

“PCA analysis revealed that the study cohort clustered predominantly with the South Asian (SAS) super-population **(Fig. 1B-i).** In the PCA with 1000 Genomes reference populations, the first two PCs explained 53.34% (PC1) and 25.42% (PC2) of the total genetic variance, respectively. A modest overlap toward the EAS cluster was observed for a subset of individuals, which is consistent with known historical admixture and regional genetic continuity within the Indian populations. These samples did not represent distinct population outliers and were therefore retained. In the within-cohort PCA, PC1 and PC2 explained 16.82% and 9.10% of the total variance, respectively. Two clear outliers were identified in this analysis and removed before imputation and downstream analyses.”

2. **Ancestry Consistency**
 The statement that the cohort was “ancestrally consistent” is reasonable based on clustering with the SAS super-population. However, since there is some overlap into EAS, please clarify whether this overlap is expected (e.g., due to regional admixture) and whether these individuals were retained or removed.

We would like to thank the reviewer for raising an important point about some overlap with the East Asian (EAS) population, and we agree that a clear explanation is necessary. We have therefore expanded the Discussion section to contextualize the observed partial overlap with the EAS super-population. The following additions have been made:

“Importantly, Indian populations are known to exhibit a complex genetic structure shaped by historical admixture between multiple ancestral components, including Ancestral North Indian (ANI), Ancestral South Indian (ASI), and East/Southeast Asian-related ancestries. Large-scale population genetic studies have consistently demonstrated gradients of East Asian-related ancestry within the SAS populations, particularly among groups from North, North-East, and Eastern India. (<https://doi.org/10.1016/j.ajhg.2013.07.006>, <https://doi.org/10.1073/pnas.1513197113>). Consequently, modest shifts of some Indian individuals towards the EAS cluster in global PCA reflect genuine population substructure rather than population misclassification or technical artefacts. Hence, these individuals were retained in the downstream analysis.”

R2-Q2: The section describing the chromosome 13 signal and the novel chromosome 2 region provides an informative overview of the mortality-specific association findings. The consistency of the Chr13 signal across two independent imputation panels is particularly notable. However, several aspects would benefit from further clarification or expansion:

1. **Statistical Strength of Associations**
 The text refers to these variants as “suggestive,” but does not state their p-values, confidence intervals, or effect sizes. Including these metrics is essential for assessing the robustness of the findings. Clarify whether any associations approach genome-wide significance or remain modest.

We appreciate the reviewer's insightful comments and have adjusted the manuscript to enhance the clarity and understanding of the mortality-specific association findings. In response to the reviewer’s comment, we have improved the Results section to explicitly report key statistical parameters for mortality-associated loci, including p-values and Odds Ratio (OR) for the chromosome 13 locus and the IndiGen-specific chromosome 2 region. These values are now referenced directly in the text in addition to their presentation in Table 2. We have further clarified that these associations remain modest and do not approach genome-wide significance.

2. **Small Mortality Sample Size**
 The mortality group (N = 66) is relatively small compared to the recovered group. Discuss how this limited sample size may reduce power, increase false-positive risk, or inflate effect size estimates.

We are grateful to the reviewer for insightful feedback. We agree that the limited mortality sample size (N=66) is an important consideration. We have now explicitly expanded the Discussion section to acknowledge the reviewer’s suggestion. The following part has been incorporated into the manuscript:

**Discussion:**

“It is important to note that the deceased cohort in this study was limited in size (N=66), reflecting the availability of real-world clinical samples collected during the first wave of the COVID-19 pandemic. This limited sample size impacts the statistical power, potential susceptibility to false-positive findings, and may inflate effect size estimates. Being conscious of this, accordingly, all mortality-associated signals identified here are interpreted as exploratory and hypothesis-generating rather than definitive genetic risk loci. The primary objective of this analysis is to prioritize ancestry-relevant regulatory regions for further investigation, with the expectation that validation in larger, independent, and ancestry-matched cohorts will be required as such datasets become available.”

3. **Population Structure Considerations**
 Since ancestry differences between recovered and mortality groups could confound associations, it would be appropriate to mention whether covariates (e.g., PCs, age, comorbidities) were included in the regression model.

We thank the reviewer for bringing these points to our attention. To address potential confounding due to ancestry or clinical heterogeneity between the recovered and deceased groups, we have clarified in the Materials and Methods section that all mortality association analyses were performed using logistic regression models adjusted for age, sex, clinical comorbidities, and population structure using the top 10 principal components (PC1-PC10). This consistent covariate framework was applied across both the imputation panels, ensuring that observed associations are not driven by uncorrected ancestry differences or demographic imbalance. The following changes have been made in the revised manuscript:

**Materials and methods: Association Testing**

“Association testing was performed using logistic regression models in PLINKv1.9, adjusting for age, sex, and comorbidities (hypertension, diabetes, cardiovascular and respiratory diseases and cancer) as covariates. **To explicitly control for population stratification and ancestry differences between the comparison groups, the top 10 principal components (PC1-PC10) derived from PCA were included as covariates in all the models.** An additive genetic model was applied to assess the association between the imputed variants and COVID-19 severity. Analyses were performed separately for: i) *Control (Mild) vs Case (Moderate + Severe)* groups and ii) *Recovered vs Deceased* patients, to evaluate the impact of host genetics on the disease progression and outcomes. **The same covariate framework (age, sex, comorbidities, and PC1-PC10) was applied consistently across both the severity and deceased analyses and across both the imputation panels.** Genome-wide significance was defined as p < 5 × 10^-8^, and variants with p < 1 × 10^-5^ were considered suggestive. Association testing was carried out separately for data imputed with the 1KGenomes and IndiGen reference panels, allowing for comparative identification of global and population-specific variants.”

R2-Q3: Interesting potential shown a regulatory locus on chromosome 2, and the integration of LD structure, genomic context, and functional plausibility is a strong aspect of the analysis. However, several points could be clarified or strengthened:

1. **Statistical Strength and Significance**
 While the LD block and suggestive SNPs are clearly described, the paragraph does not provide their p-values or effect sizes. Inclusion of these details is essential for assessing how robust and biologically meaningful these associations may be. It would also be helpful to clarify whether any SNPs approach genome-wide significance or whether the findings remain fully exploratory.

We thank the reviewer for this important suggestion. In response, we have revised the Results section to explicitly report the key statistical parameters for the chromosome 2 mortality-associated LD block, including p-values and odds ratio (OR) for the lead SNP and the linked variants. We further clarify that all variants within this LD block remain below the conventional genome-wide significance threshold (p < 5E-08) and are therefore described as suggestive associations. None of the signal’s approaches genome-wide significance, and the findings are accordingly framed as exploratory and hypothesis-generating in the revised manuscript.

2. **LD Block Interpretation**
 The LD structure is well described, but it would be useful to mention:
 – whether the LD block is unique to this study cohort or also observed in external South Asian populations (e.g., 1000G SAS).
 – whether recombination hotspots or regulatory elements are known in this region.
 This contextualization would strengthen the argument for biological relevance.

We thank the reviewer for this helpful suggestion. In response, we have added clarifying text to the revised manuscript to place the chromosome 2 LD block in an appropriate population-genetic and biological context. The following additions have been made in the revised manuscript:

“The LD block surrounding rs35575084 represents a group of tightly linked variants that is consistent with LD patterns commonly observed in the SAS populations. Indian and broader SAS populations are known to show extended LD in certain genomic regions due to shared ancestry and historical admixture. While a direct one-to-one LD comparison for this exact locus in external SAS reference datasets (such as 1000 Genomes) is limited by the sample size and allele frequency differences, the presence of a coherent LD block in our data is expected and consistent with the known population-specific LD architectures. Importantly, the relevance of this LD block is supported not by LD structure alone, but by functional annotation. The associated variants lie in the non-coding regions and overlapping regulatory chromatin marks across the endothelial, immune, and lung-related tissues, and are located near MIR4432HG, which gives rise to miR-4432. Previous studies have shown that miR-4432 regulates FGFBP1 in endothelial cells, linking this regulatory region to endothelial dysfunction, which is a key pathological feature of severe COVID-19.”

3. **Non-coding RNA Focus**
 The paragraph correctly notes that no coding genes overlap with these SNPs, suggesting regulatory roles. However, it would be valuable to discuss whether AC007100.1 or MIR4432HG show differential expression in COVID-19 or in endothelial dysfunction contexts based on existing literature or transcriptomic datasets.

We agree with the reviewer that expression context is an important consideration when interpreting non-coding loci. However, we did not find published evidence demonstrating consistent differential expression of AC007100.1 or MIR4432HG in tissues from the COVID-19 patients. Accordingly, we do not claim disease-specific transcriptional dysregulation for either of these non-coding RNAs.

Instead, our interpretation of this chromosome 2 locus is based on the convergence of multiple orthogonal lines of evidence. First, the presence of tightly linked LD block exclusively mapping to non-coding elements; second, the genomic proximity to MIR4432HG, the host gene for mir-4432 and third, proper functional studies demonstrating that mir-4432 regulates endothelial pathways through suppression of FGFBP1, given that endothelial dysfunction is a well-established feature of severe COVID-19, this functional context provides biological plausibility for the locus even in absence of COVID-19 specific transcriptomic validation.

We therefore interpret this region as a putative regulatory locus and explicitly acknowledge in the Discussion that functional validation and disease-specific expression studies would be necessary to confirm its mechanistic role.

Modified text in the discussion section is as follows:

“While direct differential expression of AC007100.1 or MIR4432HG in the COVID-19 patient tissues has not yet been reported, the locus was interpreted as a putative regulatory region based on its LD structure and prior functional evidence linking miR-4432 to endothelial biology.”

4. **Relevance to COVID-19 Pathophysiology**
The linkage to endothelial dysfunction is biologically plausible given known mechanisms of severe COVID-19. Nonetheless, adding a brief explanation of how FGFBP1 dysregulation relates specifically to vascular pathology, hyperinflammation, or thrombosis would strengthen the interpretive flow.

We thank the reviewer for this helpful suggestion. In the revised discussion, we have expanded the mechanistic interpretation of the FGFBP1 by incorporating additional literature describing its role in endothelial activation, vascular inflammation and dysregulated vascular signaling.

The revised discussion is as follows:

“FGFBP1 is expressed in endothelial cells and regulates vascular homeostasis by modulating endothelial sensitivity to vasoactive stimuli, including angiotensin II, through activation of reactive oxygen species and MAP kinase signalling pathways (<https://doi.org/10.1161/HYPERTENSIONAHA.117.10268>, <https://doi.org/10.1161/HYPERTENSIONAHA.120.15582> ).” Elevated FGFBP1 expression has been shown to promote sustained vascular inflammation, endothelial dysfunction, and heightened vasoconstrictor responsiveness in both human and experimental models (26). These processes overlap with key features of severe COVID-19, including endothelial activation and hyperinflammatory vascular signalling. Together, these observations support a biologically plausible mechanistic link between regulatory variation at the MIR4432HG locus, FGFBP1-mediated endothelial dysfunction, and COVID-19 associated vascular inflammation.”

Thank you and best wishes,

**Dr. Rajesh Pandey,** PhD

Senior Principal Scientist, CSIR-Institute of Genomics and Integrative Biology (CSIR-IGIB)

Professor, Academy of Scientific & Innovative Research (AcSIR)

Mall Road campus, Delhi-110007, India.

Professional webpage: <https://www.igib.res.in/?q=RajeshPandey>

Lab website: <https://ingen-hope.github.io/>

Google Scholar: <https://scholar.google.com/citations?user=Dj14S78AAAAJ&hl=en>

X: @RajeshPandeyLab
